# Monitoring of Air Microbial Contaminations in Different Bioenergy Facilities Using Cultural and Biomolecular Methods

**DOI:** 10.3390/ijerph16142546

**Published:** 2019-07-17

**Authors:** Elisa Anedda, Giulia Carletto, Giorgio Gilli, Deborah Traversi

**Affiliations:** Department of Public Health and Paediatrics, University of Torino, Piazza Polonia 94, 10126 Torino, Italy

**Keywords:** bioaerosol, qRT-PCR, PCR-DGGE, microbial pollution, bioenergy production

## Abstract

Bioaerosol exposure linked to the bioenergy production from waste and its effects on human health in occupational and residential environments has rising interest nowadays. The health risk associated with the exposure includes mainly infective diseases, allergies, chronic bronchitis, and obstructive pulmonary disease. A risk assessment’s critical point is the bioaerosol quality and quantity characterization. The aim of this study is to evaluate the application of different methods for the analysis of bioaerosol sampled in bioenergy plants. This study involved six Italian plants for the treatment of biomasses and energy production. Bioaerosol cultural evaluation was performed, by Surface Air System (SAS) sampler, and DNA was extracted from PM0.49 samples and Low Melting Agar plates. qRT-PCR followed by Denaturing Gradient Gel Electrophoresis (DGGE) and band sequencings were performed. The cultural method is able to detect less than 15% of what is evaluable with bio-molecular methods. A low sample alfa-diversity and a high beta-biodiversity in relation to feedstock and process were observed. Sequencing showed microorganisms with a hygienic-sanitary relevance such as *Arcobacter*, *Pseudomonas*, *Enterobacter*, *Klebsiella*, *Enterococcus* and *Bacillus*. Integrated cultural and biomolecular methods can be more exhaustive to evaluate bioaerosol’s exposure in the occupational environment.

## 1. Introduction

Nowadays, bioenergy production aims to contribute towards preservation and restoration of the environment quality in terms of pollution to safeguard human health. Bioenergy can be produced from various biomasses, such as organic fraction of municipal solid waste (OFMSW), agricultural and livestock, waste-water sludge, and lignocellulose wastes. The major attention on waste differentiation and disposal is towards increasing the organic material addressed to specific treatment, leading to bio-refineries development [1]. The number of workers involved increases consequentially [2] and biomasses transformation processes are receiving great attention, especially considering the potential bioaerosol exposure and its health effects [3]. Biological risks, associated with biological agents and bioaerosols, were generally underestimated in occupational settings thought history [2,4]. Bioaerosol consists of viable or non-viable airborne microorganisms like bacteria, fungi, virus, protozoa, algae, pollen, endotoxins, and others [5,6]. Due to their characteristics, bioaerosols are able to remain suspended in the air for prolonged periods of time and potentially travel long distances from their emission source and it may result in health effects for workers and nearby communities [4]. On the other hand, biological mixtures represent a major risk potentially associated with acute and chronic adverse health effects and diseases such as asthma, rhinitis, sinusitis, and bronchitis [7,8,9]. Other health problems include fatigue, weakness, and headaches [7]. The respiratory tract contains microbial communities having a major role in the maintenance of human health and those communities can inhibit the pro-inflammation process caused by pathogenic microorganisms [10]. Bioaerosol composition, individual exposure, and health effects are extremely variable and not yet completely known, therefore the definition of exposure limits for bioaerosol and its effect on human health are still unfeasible [2,11]. Occupational exposure limits to bioaerosol, proposed at local levels, include mainly mesophilic bacteria, endotoxins, and fungal spores [12]. A risk assessment’s critical point process is the characterization step where the bioaerosol quality and quantity are crucial data to correlate to human health damage [2,13,14]. There is still a lack of evidence on the human dose/response relationship and on standard protocols to adopt for bioaerosol sampling and analysis [15]. The increase and heterogeneity of the biomasses involved in biofuel production make the biological risk assessment a priority in the plants. The cultured method for bioaerosol analysis is generally used in routine monitoring, however it is clear that such method produces an underestimation of the real contamination. Biomolecular methods are able to produce a picture of the airborne microbiota useful to assess biological risk, especially for the higher method sensibility [16,17]. Specific attention to pathogens and opportunistic pathogens presence and persistence in occupational framework is a necessity in terms of homogenization of knowledge and evaluation methods in order to safeguard workers health [18]. The sampling includes various methods generally with a low/medium flow sampling rate. Concerning air pollution monitoring, one of the most validated procedures is the inhalable particles matter samplings, that can be conducted with higher flow rate producing concentrated extracts.

The aim of this study is to evaluate the application of different techniques for sampling and analyzing the bioaerosol contamination in various bioenergy plants. The sampling includes both microbiological plates and filters while the analysis include both cultural and biomolecular methods. Finally, the aim is to gain knowledge on bioaerosol composition and hazards in different bioenergy plants.

## 2. Materials and Methods

### 2.1. Plant Descriptions

The plants considered in this work are bioenergy plants utilizing different kinds of biomasses: agricultural and livestock biomasses (ALB), wastewater treatment sludge (WWTS), the organic fraction of municipal solid waste (OFMSW), and lignocellulosic biomasses (LCB). All the plants were located in Piedmont and the first three were previously described [19]. Agricultural residues such as *Arundo donax*, rice straw, or wheat straw were used as lignocellulosic biomass in the LCB plant. Plants applied mainly anaerobic digestion for biomethanization. In the LCB plant, the final energy vector is bioethanol.

#### Sampling Points

The sampling was conducted at least two times in two different areas of each plant following international standards [20,21]. In the two ALB, WWTP, and OFMSW plants, sampling was conducted in the area closed to the feedstock introduction into the anaerobic digester and in the area where the effluent sludge came out. Into the LCB plant, the sampling was conducted in the receiving area of the lignocellulosic biomasses and in the area where the partially dewatered stream, consisting mainly of raw lignin portion, unconverted feed, and other by-products, accumulated and then headed to disposal.

### 2.2. Sampling Methods

#### 2.2.1. Particulate Matter (PM) < 0.49 Extraction

Sub-fractionated PM10 was sampled in the six plants between August 2016 and July 2017. In each plant, at least two different sites were chosen as sampling sites (“loading” and “output”) where the occupational exposure could be relevant. Each sampling was performed for a maximum of 4 h. PM was collected using a high-volume cascade impactor (the AirFlow PM10-HVS sampler is a multi-stage cascade impactor, with pre-selectors complying with the UNI EN-12341 norm, by Analitica Strumenti, Pesaro, Italy) at an electronically controlled flow of 1.27 m^3^/min. The particle size fractions were as follows: 10.0–7.2, 7.2–3.0, 3.0–1.5, 1.5–0.95, 0.95–0.49, and <0.49 μm. Only glass microfiber filters (203 × 254 mm; Pall Corporation, Washington, NY, USA) were used to collect the finest particles (<0.49 μm) and were processed for biomolecular analyses.

#### 2.2.2. Bioaerosol Sampling

Bioaerosol sampling was performed using a DUO Surface Air System (SAS) Super 360 sampler (PBI International, Milan, Italy), which allows microbial monitoring through air contact on apposite Petri plates (RODAC™ Contact Plates, VWR, Radnor, Pennsilvanya, USA). For cultural analyses, the microbiological parameters were bacterial 22 °C count, bacterial 37 °C count and bacterial 55 °C count. The plates were prepared in the laboratory a few days before the sampling, following the medium instructions as previously described [19]. For biomolecular analyses, plate preparation was performed as previously described [22,23] and on each plate 2000 L of air was sampled. Low Melting Agarose (LMA) (Ultra-Pure™ LMP Agarose 16520-050, Invitrogen, Carlsbad, California., USA) was prepared with deionized water following the product instructions. A total of 4 mL of medium were transferred on each Petri plate. After sampling, the LMA was transferred in sterile Falcon with a sterile spatula, then stored at −80 °C prior to extraction. The LMA was used as device of capture, not as cultural substrate.

### 2.3. DNA Extraction and Real-Time PCR

DNA extraction protocol was the same for filters and for LMA and was performed using Power Viral Environmental RNA/DNA kit (#28000-50 Qiagen, Hilden, Germany):

- For filter extraction (24 total samples) 1/16 of glass microfiber filter (corresponding to 19 m^3^ of sampled air) was used. Filters are made of porous material and in the first step of extraction, more lyses buffer was used (2 mL);
- For LMA extraction (32 total samples) the agarized medium was depolymerized by a microwave for a few seconds; the extraction was performed starting from 200 µL, corresponding to 0.1 m^3^ of sampled air.

In Table 1 the sample description is reported. After genomic extraction, the nucleic acids were quantified using a Nano Quant Plate™ (Tecan Trading AG, Switzerland) which allows the quantification through a spectrophotometer read at 260 nm. The spectrophotometer used was Tecan Infinite^®^ 200 PRO and the software was i-control™ (version 1.11.10). The protein and lipid contamination indices were calculated through two ratios, A260/A280 and A260/A230 respectively, to certify the purity of the extract. The average DNA extracted concentration from filters was 26.21 ± 7.85 ng/L and from LMA, 1.05 ± 0.70 ng/L.

Real-time PCR was performed, with the CFX Touch System (Bio-Rad, Hercules, California, USA), to quantify total bacteria. Standard curves were produced using serial seven-fold dilutions of purified *Desulfovibrio vulgaris* DNA starting from a concentration of 1.52 × 10^6^ gene copies. The reaction was set as follows: 95 °C (3 min), 39 cycles of 95 °C (10 s), 59 °C (15 s), 72 °C (10 s), and 72 °C for 5 min and a final melting curve step of 0.5 °C/cycle for 60 cycles at 65 °C (31 s) and then 65 °C (0.05 s). The reaction was performed using iQ™ Multiplex Powermix (BioRad, USA).

### 2.4. PCR-DGGE and Sequencing

For all the samples, PCR was performed using primer pair specific for 16 S rRNA genes of bacteria (Table 2). PCR was performed using Master mix for PCR (Bio-Rad, USA) with addition of Bovine Serum Albumin (BSA) to enhance the reaction efficiency [24] with primers 357FGC/518R for Bacteria. Samples were stored at −20 °C prior to DGGE. DGGE gel was produced in order to obtain a denaturing gradient of urea and formamide from 30% to 50% [25] using 40% Acrylamide/Bis-Acrylamide, formamide, urea (Sigma-Aldrich, St. Louis, Missouri, USA), and TAE50x. Ammonium persulfate (APS) and Tetramethyl ethylenediamine (TEMED, Sigma-Aldrich) were added to 30% and 50% solutions to catalyze acrylamide polymerization. A gradient was created using a gradient maker located on a magnetic mixer and connected to a peristaltic pump. The marker was composed by 16S amplificates from genomic DNA of *Geobacter metallireducens* (ATCC^®^ 53774D-5) and *Shewanella oneidensis* (ATCC^®^ 700550D). The electrophoretic run was performed using the DCode System for DGGE by Bio-Rad for 5 h at 200 V. Gel was stained with SYBR Green I (Sigma-Aldrich) and bands were observed with the transilluminator Gel Doc XR (Bio-Rad). Bands were cut and purified with sterile water prior the reamplification, which was performed using the same primer pairs, with the addition of an M13 tail to the reverse primer as described in the literature [26] (Table 2). Reamplification was performed with the same mix used for previous PCR, without BSA and the conditions were as follows: 72 °C (10 min), 10 cycles of 94 °C (30 s), 55 °C (30 s) and 72 °C (1 min), 25 cycles of 92 °C (30 s), 52 °C (30 s) and 72 °C (1 min), and final step at 72 °C (10 min). Reamplified samples were delivered to an external laboratory (Ylichron Srl Genechron Srl) for Sanger sequencing.

### 2.5. Data Analysis and Statistics

All the samples were tested in triplicate under good laboratories practice and procedural reference. Sequenced samples were analyzed with Finch TV (v. 1.4.0) and BLASTn to identify microorganisms. Bionumerics 7.6 was used to analyze DGGE images, for taxonomic assignment corresponding to sequencing, to calculate the Shannon’s diversity index and to build dendrograms. Clustering was performed with Pearson’s correlation index and dendrograms were created with unweighted pair group method with arithmetic mean (UPGMA). Statistical analyses were performed using the SPSS Package, version 24.0 (IBM Corp., Armonk, New York, USA). We applied (1) a log transformation to non-normally distributed data, (2) the Spearman’s correlation to assess relationships between variables, (3) *t*-test and paired *t*-test (where appropriate) to compare means and (4) ANOVA for multivariate analysis followed by a Tukey post-hoc test for multiple comparisons. The mean differences and correlations were considered significant for *p* < 0.05 and highly significant for *p* < 0.01. 

## 3. Results and Discussion

### 3.1. Cultural Method vs. Biomolecular Method

The first phase of our study was focused on the comparison between the cultural method and biomolecular method for airborne bacterial community composition. In Table 3 the cultural microbiological analysis and total biomolecular mesophilic bacteria count are briefly summarized. LCB plants showed a higher concentration of total bacteria with all three methods compared to other plants (ANOVA, *p* < 0.05). 

**Figure 1:**
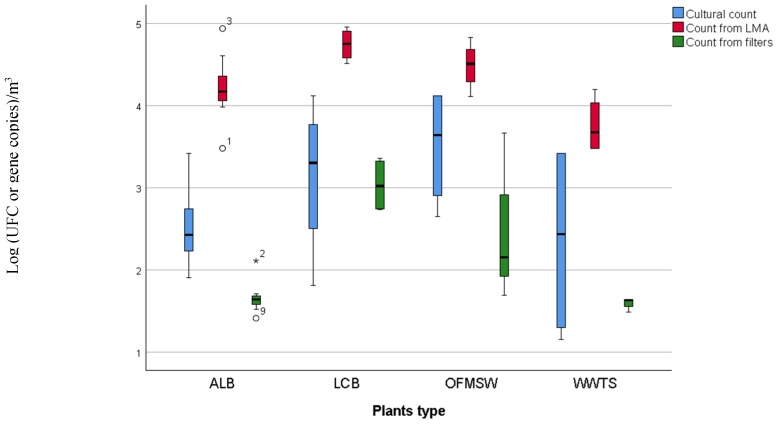
Boxplot of bacterial quantification methods for each plant type. Legend: cultural count represents the total mesophilic cultural count (37 °C, UFC/m^3^), count from LMA represents the biomolecular quantification with DNA extracted from LMA plates (copies/m^3^) and count from filters represents the biomolecular quantification with DNA extracted from filter (copies/m^3^).

In Figure 1 cultural mesophilic bacteria count and total biomolecular bacteria quantification, in relation to plant type, are reported. The cultural method, using plate count agar, resulted as a good technique more sensitive than bacteria evaluation from PM0.49 filters. On the other hand, it allowed investigating only around 15% of what was observed on biomolecular quantification from LMA plates. Therefore, cultural methods are useful for first screening analysis of bioaerosol, but appears a scarce, sensitive technique for biological risk evaluation. In fact, bacteria quantification from LMA resulted as higher for all plants type, thus suggesting that low melting agar is a good medium for airborne bacteria and subsequent biomolecular analyses. Furthermore, filters resulted as the worst method for bacteria quantification and despite a higher DNA concentration obtained from extraction, bacteria quantification was poor and a 260/230 ratio may suggest carbohydrate contamination (<2.2) and low DNA quality. This could be due to not optimal extraction methods to the high dehydration during high flow PM0.49 sampling. In addition, total bacteria quantification significantly correlated with DNA concentration from LMA (Spearman’s rho = 0.487; *p* < 0.01), but this correlation was not significant for the DNA extracted from filters. Eventually, biomolecular quantification with real-time PCR allows quantifying uncultured bacteria but it might overestimate the 16 S genomic units that do not necessarily correspond to vital cells.

### 3.2. Biodiversity Analysis

**Figure 2:**
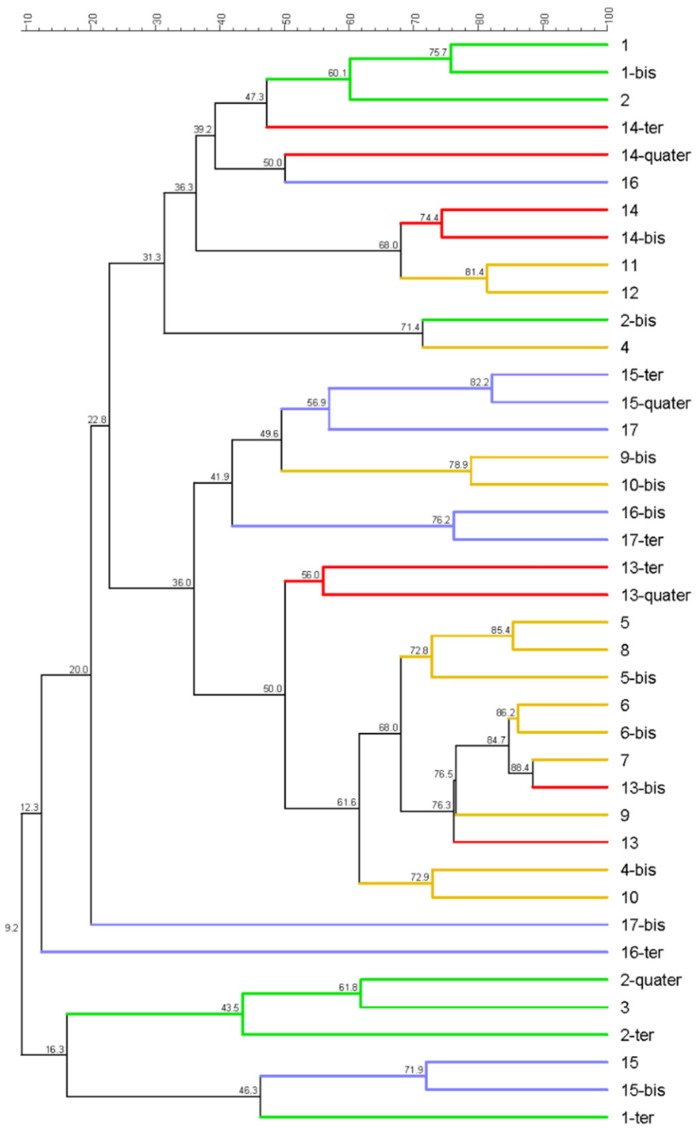
Phylogenetic analysis, bacteria dendrogram of samples. Pearson similarity coefficients are reported near the node. Colour legend: green (organic fraction of municipal solid waste (OFMSW)), yellow (agricultural and livestock biomasses (ALB)), red (wastewater treatment sludge (WWTS)), and violet (lignocellulosic biomasses (LCB)). Replicate samples (i.e., 1-1bis-1ter-1quater) are also reported. Whenever -ter and -quarter replicates are present, those are from LMA samples.

**Figure 3:**
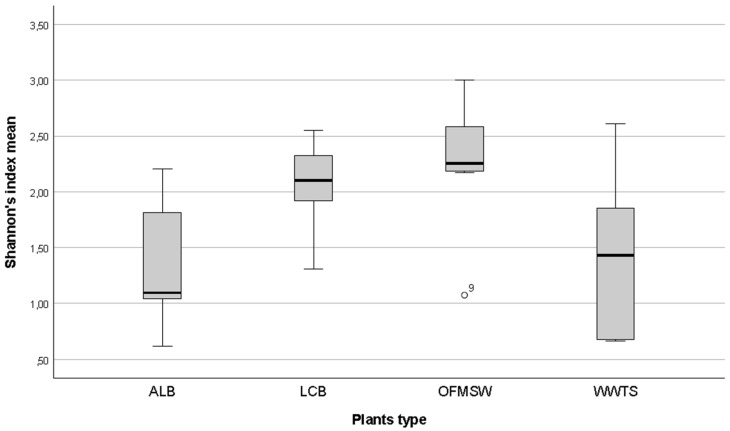
Boxplot of biodiversity Shannon’s indices in relation to the type of plant investigated.

Out of 56 samples, only 40 showed DGGE-bands and they came mostly from PM0.49 filters (26 vs. 14). This was probably due to the higher air volume (see Section 2.3) and also to the higher amount of biological materials collected on filters by PM sampling. However, no significant correlation between DNA concentration (ng/L) and band presence was found, neither a correlation between DNA ng/L and type of plant. As shown in Figure 2, by phylogenetic cluster, the samples mainly clusterized in relation to the type of plant and a high heterogeneity and biodiversity between samples was detected. Moreover, whenever biodiversity was higher, it resulted as similar (>80% similarity) between pairs of replicates. Repeated sampling in different areas of the same plant showed different results and a wider variability, confirming the bioaerosol and biomass complexity. In Figure 3 Shannon index means are reported. As expected, a wide diversity in relation to the types of feedstock was observed. OFMSW and LBC plants were different from the others (Shannon’s indices 2.27 and 2.08 respectively). ALB and WWTP showed a higher evenness and similarity (Shannon’s indices 1.33 and 1.40 respectively).

Despite the different DNA concentration extracted from LMA and filters, similar biodiversity was observed (Shannon’s index = 1.7), suggesting that biodiversity is not affected by the sampling method. In the future, NGS application methods could reduce the complication due to the high-resolution limit of the DGGE-technique, even if, to date not so more informative data on such environmental samples are currently produced [28].

Shannon’s index significantly correlates with bacterial count at 37 °C (Spearman’s rho = 0.43; *p* < 0.05), bacterial count at 55 °C (Spearman’s rho = 0.565; *p* < 0.01), and total bacteria gene copies (Spearman’s rho = 0.348; *p* < 0.05).

### 3.3. Sequencing Analysis

Bands were selected for sequencing, and results showed various profiles with a high hygienic-sanitary relevance. The profiles belonged to the families of *Campylobacteraceae*, *Pseudomonadaceae*, *Bacillaceae*, *Enterobacteriaceae*, *Comamonadaceae*, and *Burkholderiaceae*. In particular, *Arcobacter skirrowii, Pseudomonas fluorescens*, *Pseudomonas protegens*, *Enterobacter* spp., *Enterobacter cloacae*, and *Comamonas sp.* Additionally, Firmicutes such as *Enterococcus* spp. and *Lactobacillus* sp. were found, as summarized in Table 4. Moreover, various uncultured bacteria were found, as also reported in the literature [28,29,30,31]. Among the uncultured bacteria, *Ralstonia* spp., *Bacillus* spp., and *Klebsiella* spp. were found and they were primarily identified in environmental samples, food waste, and influent wastewater, respectively. Uncultured *Bacillus* spp. was found in input samples of the OFMWS plant and this data confirmed the microbial contamination analysis of the air previously reported [19]. Moreover, *Pseudomonas* spp. was identified in output samples of the OFMWS plant, confirming previous microbial analysis, as well. In addition, LCB showed *Pseudomonas* spp. in the input sample. *Bacillus* spp. and Pseudomonadaceae were typically associated to biofilm maturation with subsequent biofilm dispersion [32,33]. Uncultured *Klebsiella* spp. were found in input sample of the OFMSW plant. *Klebsiella* spp. as well as *Pseudomonas* spp. are known antimicrobial resistant genera, in particular the species *Klebsiella pneumoniae* and *Pseudomonas aeruginosa* representing a major hygienic emergency throughout Europe [34]. The Firmicutes phylum, in particular the genera *Clostridium* and *Bacillus*, may represent great indicators for biological contamination for both aerobic and anaerobic environments [35], typical of bioenergy plants. Both genera can cause diseases frequently linked to their protein toxins and spores [35] and the identification and quantification may represent a first step for their monitoring in biomethanization plants and for the safeguard of workers health.

Furthermore, *Arcobacter skirrowii* was found in output samples of both OFMSW and LCB plants, suggesting a fecal pollution of the environment and a potential source of infection [36]. *Arcobacter skirrowii* could be antimicrobial resistant as well as other two, *A. butzleri* and *A. cryaerophilus* [37], and further studies could be crucial to better understand its role in human disease. *Enterobacter cloacae*, a microbial resistant fecal coliform, was found in the input sample of OFMSW and this discovery is consistent with the literature [3]. Uncultured *Enterococcus* sp. was found in the input sample of the OFMSW plant, confirming previous microbial analysis that identified *E. faecium* and *E. faecalis* [19]. These species are intrinsically resistant to a broad range of antimicrobial agents and are generally associated with a variety of infections [34]. Furthermore, uncultured *Ralstonia* spp. was found in input and output samples of WWTS and ALB plants. Despite the lack of data regarding *Ralstonia* spp. findings in such environments, *Ralstonia pickettii* was identified in both influent and effluent samples of an anaerobic bioreactor that was treating cattle manure [38]. Moreover, despite high DNA concentrations of ALB samples, the sequencing resulted in few bacteria identification probably due to outdoor and semi-outdoor environment and a high fungi contamination, as previously reported [19]. In such plants a dilution effect occurs [39] therefore a massive sampling and the acquisition of more genomic material are feasible, but less specific in relation to bacterial DNA.

### 3.4. Strong Point and Limitations

In the present work the sampling also on filters by high flow rate sampler commonly used for PM10 monitoring was conducted. This is a novel method, on the other hand the extraction procedure from filters probably have to be optimized. The application of biomolecular methods allows a better characterization of the bioaerosol in bioenergy plants showing that in such environment the contamination level, suggested as occupation limits in several country from 10^3^ to 10^4^ UFC/m^3^, are often reached [15]. This supports the necessity of an accurate biological risk assessment for each worker profile.

The application of NGS technology, into biomolecular analysis, will provide additional knowledge in the future, however the first literature data in this field are not yet able to produce clear innovative evidence. Moreover, such approach is far from the integration into the monitoring and assessment produced by the plants, even if the evolution of the bioenergy plant is very rapid and consistent including new professional and technical skills.

## 4. Conclusions

Our results suggested that cultural methods cannot be exhaustive to evaluate biological risk in occupational environment due to the matrices of complexity and heterogeneity. On the other hand, it could be a useful method for qualitative analyses of the environment. The biomolecular methods, such as quantitative PCR, could be useful especially starting from an LMA plate instead of filter. An additional biomolecular analysis, such as PCR-DGGE, seems a low-cost method for a screening evaluation of the airborne microbiome and to understand which microorganisms are most frequently detected in such biomasses. 

The known-to-date methods are mostly able to give a coarse description, but the standardization of a quantitative method for bioaerosol composition seems to be crucial. Moreover, single sampling could not be truly representative of the actual microbiome composition and it is not known how microorganisms’ concentrations may vary with longer periods of exposure. Therefore, creating a protocol for complex matrices samplings is desired, that specifically considers time of exposure and microbial variation through time. The study also opened future biomolecular perspectives for a better characterization using NGS methods for example, and microbial identification, including fungi and virus. In fact, it may enhance the characterization of the microbiome, despite the well-known disadvantages of the method such as cost and weak standardization, especially for bioinformatic analysis. Furthermore, the identification of bioindicators such as *Bacillus* and *Clostridia* genera may be useful for contamination and health risk assessment associated with its exposure.

## Figures and Tables

**Table 1:** Description of the collected samples: plant type, number of filters (<0.49 µm), and number of low melting agar plates.

Plant Type	N° Filters	N° LMA Plates
**OFMSW**	5	4
**ALB**	17	12
**WWTS**	4	4
**LCB**	6	4

**Table 2:** Primers used for PCR-DGGE, DGGE band re-amplification and for total bacteria quantification.

Primers	Sequence (5′−3′)	Target Genes	References
**357F-GC^a^**	GCclamp -CTC CTA CGG GAG GCA GCA G	16S rRNA Bacteria	[25]
**518R**	GTA TTA CCG CGG CTG CTG G		
**357F-GC**	GCclamp -CTC CTA CGG GAG GCA GCA G	16S rRNA Bacteria	[25,26]
**518R-AT-M13**	GTAAAACGACGGCCAGTAAATAAAATAAAAATGTAAAAAAATTACCGCGGCTGCTGG		
**TotBact F** **TotBact R**	ACTCCTACGGGAGGCAGCAG ATTACCGCGGCTGCTGG	16S rDNA Total Bacteria	[27]

**Table 3:** Bacterial count data summarized in relation to plant type: mean and standard deviation of bacterial quantification from Low Melting Agarose (LMA) and filters and total mesophilic bacteria count.

Plant Type	LMA Bacterial Count Log10 (gene copies/m^3^)	Total Count at 37 °C Log10 (UFC/m^3^)	Filter Bacterial Count Log10 (gene copies/m^3^)
**ALB**	4.27 ± 0.35	2.49 ± 0.44	1.65 ± 0.16
**LCB**	4.75 ± 0.20	3.14 ± 0.97	3.03 ± 0.33
**OFMSW**	4.41 ± 0.31	3.18 ± 0.97	2.42 ± 0.86
**WWTP**	3.76 ± 0.35	2.36 ± 1.23	1.60 ± 0.07

**Table 4:** Taxonomic assignment of sequencing reads from the bacteria community (16S rRNA) of bioaerosol samples for each plant type.

Plants	Area	Length	Closest Relative	Accession N°	%	Phylum	Order	Family	Genus
OFMWS	Input	160	*Enterobacter* sp.	GQ465229	95	Proteobacteria	Enterobacterales	*Enterobacteriaceae*	*Enterobacter*
		159	*Enterobacter cloacae*	KX303810	98	Proteobacteria	Enterobacterales	*Enterobacteriaceae*	*Enterobacter*
		160	Uncultured *Bacillus* sp.	KM819125	94	Firmicutes	Bacillales	*Bacillaceae*	*Bacillus*
		158	Uncultured *Enterococcus* sp.	LT625552	91	Firmicutes	Lactobacillales	*Enterococcaceae*	*Enterococcus*
		160	*Lactobacillus* sp.	KC755084	100	Firmicutes	Lactobacillales	*Lactobacillaceae*	*Lactobacillus*
		158	Uncultured *Klebsiella* sp.	LC342860	90	Proteobacteria	Enterobacterales	*Enterobacteriaceae*	*Klebsiella*
	Output	135	*Arcobacter skirrowii*	MG195899	100	Proteobacteria	Campylobacterales	*Campylobacteraceae*	*Arcobacter*
		160	*Pseudomonas fluorescens*	EU434416	94	Proteobacteria	Pseudomonales	*Pseudomonadaceae*	*Pseudomonas*
		160	*Pseudomonas protegens*	MG269715	100	Proteobacteria	Pseudomonales	*Pseudomonadaceae*	*Pseudomonas*
		160	*Comamonas* sp.	JF928565	100	Proteobacteria	Burkholderiales	*Comamonadaceae*	*Comamonas*
ALB	Output	160	Uncultured *Ralstonia* sp.	MG801673	100	Proteobacteria	Burkholderiales	*Burkholderiaceae*	*Ralstonia*
WWTS	Input	160	*Comamonas* sp.	JF928565	100	Proteobacteria	Burkholderiales	*Comamonadaceae*	*Comamonas*
		160	Uncultured *Ralstonia* sp.	MG801673	98	Proteobacteria	Burkholderiales	*Burkholderiaceae*	*Ralstonia*
LBC	Input	160	*Pseudomonas* sp.	MH394449	100	Proteobacteria	Pseudomonales	*Pseudomonadaceae*	*Pseudomonas*
	Output	135	*Arcobacter skirrowii*	MG195900	100	Proteobacteria	Campylobacterales	*Campylobacteraceae*	*Arcobacter*
